# Disruption of Amyloid Plaques Integrity Affects the Soluble Oligomers Content from Alzheimer Disease Brains

**DOI:** 10.1371/journal.pone.0114041

**Published:** 2014-12-08

**Authors:** Sebastian Jimenez, Victoria Navarro, Javier Moyano, María Sanchez-Mico, Manuel Torres, Jose Carlos Davila, Marisa Vizuete, Antonia Gutierrez, Javier Vitorica

**Affiliations:** 1 Departamento Bioquímica y Biología Molecular, Facultad de Farmacia, Universidad de Sevilla, Sevilla, Spain; 2 Instituto de Biomedicina de Sevilla (IBiS)-Hospital Universitario Virgen del Rocío/CSIC/Universidad de Sevilla, Sevilla, Spain; 3 Departamento Biología Celular, Genética y Fisiología, Facultad de Ciencias, Instituto de Investigación Biomédica de Málaga (IBIMA), Universidad de Málaga, Málaga, Spain; 4 Centro de Investigación Biomédica en Red sobre Enfermedades Neurodegenerativas (CIBERNED), Madrid, Spain; IIBB/CSIC/IDIBAPS, Spain

## Abstract

The implication of soluble Abeta in the Alzheimer’s disease (AD) pathology is currently accepted. In fact, the content of soluble extracellular Abeta species, such as monomeric and/or oligomeric Abeta, seems to correlate with the clinico-pathological dysfunction observed in AD patients. However, the nature (monomeric, dimeric or other oligomers), the relative abundance, and the origin (extra-/intraneuronal or plaque-associated), of these soluble species are actually under debate. In this work we have characterized the soluble (defined as soluble in Tris-buffered saline after ultracentrifugation) Abeta, obtained from hippocampal samples of Braak II, Braak III–IV and Braak V–VI patients. Although the content of both Abeta40 and Abeta42 peptides displayed significant increase with pathology progression, our results demonstrated the presence of low, pg/µg protein, amount of both peptides. This low content could explain the absence (or below detection limits) of soluble Abeta peptides detected by western blots or by immunoprecipitation-western blot analysis. These data were in clear contrast to those published recently by different groups. Aiming to explain the reasons that determine these substantial differences, we also investigated whether the initial homogenization could mobilize Abeta from plaques, using 12-month-old PS1xAPP cortical samples. Our data demonstrated that manual homogenization (using Dounce) preserved the integrity of Abeta plaques whereas strong homogenization procedures (such as sonication) produced a vast redistribution of the Abeta species in all soluble and insoluble fractions. This artifact could explain the dissimilar and somehow controversial data between different groups analyzing human AD samples.

## Introduction

Progressive aggregation and accumulation of extracellular amyloid-β (Abeta) peptides is central to Alzheimer’s disease (AD) pathogenesis [6]. Multiple Abeta oligomers, ranging from low-molecular weight oligomers to protofibrils and fibrils, apparently coexist in the brain tissue. However, the exact nature of these toxic aggregated Abeta peptides and their origin (intra- or extraneuronal or even plaque-derived) remains speculative. After the initial investigation by Podlisny et al. [15], a growing number of evidences strongly indicate that soluble Abeta forms, rather than insoluble species, including amyloid plaques, are the main toxic species associated with AD (see [1] and [5], for recent reviews). Subsequent studies by different independent groups, in an attempt to identify the precise soluble form that best correlated with cognitive impairment, have in fact reported a remarkable diversity of soluble aggregates in the interstitial fluid of human AD and transgenic model brains. Based on *in vitro*, using synthetic Abeta peptides, or “*in vivo”* experiments, different soluble Abeta oligomers, ranging from dimers to high-molecular weight aggregates (such as 12-mer, 24-mer, 32-mer, 150-mer) have been identified as the putative neurotoxic agents, disturbing the neurotransmission and causing neuronal death (see table 1 from [1]). The reasons for this apparent heterogeneity are not known but could derive from the different aggregation protocols of synthetic Abeta (for instance see [2] and [11]), the different transgenic models used or the different brain regions characterized. In this sense, it is particularly intriguing the quantitative and qualitative differences between soluble Abeta isolated by microdialysis and by homogenization-centrifugation approaches, reported even by the same group [24].

**Table 1:** Human samples.

mean Age ± SD	Sex	Delay post-mortem mean h ± SD	BRAAK
84.0±5.47	7 male - 4 female	6.2±4.5	II
78.1±11.99	4 male - 3 female	5.76±5.44	III–IV
80.4±10.21	4 male - 8 female	11.2±4.0	V–VI

Perhaps more relevant is the fact that, using brain samples from Alzheimer patients, a great disparity in the nature and quantities of soluble Abeta has been observed. For instance, relatively large amounts of monomeric and dimeric soluble Abeta (identified by western blots) have been reported by some groups [3], [12], [13], [17], [23], [24] whereas others found small amounts (pg range, detected by dot blots or sensitive oligomeric specific ELISA assays) of soluble Abeta [4], [21]. Among multiple causes, these differences between groups could reflect the absence of a unified isolation protocol for the extraction and characterization of soluble Abeta. In fact, many different homogenization (from manual to sonication) and centrifugation protocols have been used to isolate a theoretically similar extracellular soluble fraction. Considering that Abeta plaques constitute a large reservoir of different Abeta species, and that their number increased with the pathology, strong homogenization protocols could indeed disturb the stability of plaques, and cause the release of different Abeta species. However, few (if any) control experiments were carried out to determine the extent of plaque disruption due to the isolation procedure.

In the present study, we analysed the Abeta content in the soluble fractions of the hippocampus from human AD autopsy samples and transgenic models in order to investigate the possible repercussion of the homogenization protocols in the soluble fraction preparation. For that, we have directly compared the effect of homogenization using gentle conditions with sonication at different intensities. We concluded that strong homogenization, such as sonication, produced the release of Abeta peptides from Abeta plaques to soluble fractions.

## Materials and Methods

### Antibodies

82E1 and anti-soluble APPalpha antibodies were purchased from Immuno Biological Laboratories (IBL). 6E10 antibody was provided by Signet Laboratories. Anti-LC3B and anti-ATP synthase-beta were purchased from Cell Signaling Laboratories and BD Transduction Laboratories, respectively. Anti-mouse or anti-rabbit horseradish-peroxidase-conjugated secondary antibodies were purchased from Dako Denmark.

### Human samples

The study was performed using 30 cases obtained from the BTIN-Tissue Bank for Neurological Research (Madrid, Spain) and from the Neurological Tissue Bank of IDIBELL-Hospital of Bellvitge (Barcelona, Spain), approved by the committee for human and animal use for research at Seville and Malaga Universities, Spain. The cases were scored for Braak stage for neurofibrillary tangles (II–VI). In Table 1 are detailed the age, Braak stage, sex and the delay post-mortem before the extraction of the samples.

### Preparation of human brain lysates

Soluble (S1) fractions were prepared as described previously [9], [10], [19], [22]. Briefly, frozen human hippocampal tissue was homogenized (1/5 w/v) using a manual Dounce homogenizer (10 strokes using pestle A (large clearance: 0.114±0.025 mm) and 10 strokes using pestle B (small clearance: 0.05±0.025 mm), in Tris-buffered saline (TBS; 20 mM Tris-HCl, 140 mM NaCl, pH 7.5) containing protease and phosphatase inhibitors (Roche). Homogenates were then ultracentrifuged (4°C during 60 min) at 100,000×g (TLA110 rotor, Optima MAX Preparative Ultracentrifuge, Beckman Coulter). The supernatant, which constitutes the S1 fractions, was collected, aliquoted and stored at −80°C for further use. The remaining pellet (P1) was stored at −80°C until needed.

### Sequential protein extraction

For sequential extraction experiments, the P1 fraction was first resuspended (by Dounce homogenization; 10 strokes using pestle B) in RIPA buffer (1% CHAPS, 1% deoxycholate, 0.2% SDS, 140 mM NaCl, 10 mM Tris-HCl, pH 7.4, containing protease and phosphatase inhibitors), incubated for 30 min at 4°C with agitation, and centrifuged at 30,000×g for 30 min at 4°C. The supernatant constituted the S2 fraction. The pellet from this centrifugation was sequentially extracted (by incubating during 30 min, 4°C followed by centrifugation 30,000×g for 30 min) using buffered-SDS 2% (2% SDS in 20 mM Tris-HCl, pH 7.4, 140 mM NaCl) (S3 fraction) and 4% SDS plus 8 M urea (P3 fraction). All isolated fractions were aliquoted and stored at −80°C until use.

### Abeta quantification by sandwich ELISA

The amount of soluble Abetax-40 or Abetax-42 peptides was determined in human soluble S1 fractions, using commercial sandwich ELISA (Human Abx-40 ELISA Kit, Invitrogen; Amyloid beta x-42 ELISA, DRG) following the manufacturer recommendations. For these experiments equivalent amount of proteins, from the different S1 fractions isolated from Braak II to Braak V–VI samples, were pooled. For each assay, 25 µg of protein from the pooled-soluble fractions was used. The ELISA experiments were repeated four times, in independent experiments, using duplicate or triplicate replicas.

For oligomeric ELISA, we used 6E10-6E10 antibody pairs in conjunction with the Alpha-Lisa technology, developed by Perkin Elmer. This assay recognized synthetic Abeta dimer, used as standards, whereas synthetic monomeric Abeta42 produced no signal over the background. The lower limit sensitivity of this assay was 0.02 pg/µl.

### Preparation of PS1delta9xAPPswe cortical fractions

All animal experiments were performed in accordance with the guidelines of the Committee of Animal Research of the University of Seville (Spain), University of Malaga (Spain) and the European Union Regulations.

B6.Cg-Tg(APPswe,PSEN1dE9)85Dbo/J (PS1xAPP) transgenic model was maintained in C57BL/6 genetic background for several generations (F10). Cortical samples from 12 months PS1xAPP transgenic mice were homogenized in TBS (1/10 w/v) containing protease and phosphatase inhibitor-cocktail (Roche), using two different approaches: a) manual Dounce homogenizer (10 strokes with pestle A and 10 strokes with pestle B) or b) sonication (100 W ultrasonic processor UP100 H, Hielscher) for x4 or x8, 15 sec pulses at 4°C.

After homogenization, the different fractions were isolated as described in [17]. Briefly, homogenates were first centrifuged at 14,000×g (30 min, 4°C). The supernatant (containing soluble and microsomal fraction) was then ultracentrifuged at 100,000×g (60 min, 4°C) using a TLA110 rotor (Optima MAX Preparative Ultracentrifuge, Beckman Coulter). The supernatant of this ultracentrifugation constituted the TBS soluble fraction. The pellet, that constituted the microsomal fraction, was resuspended in TBS.

On the other hand, pellets from the first centrifugation (membranes plus Abeta plaques) were sequentially solubilized using 2% buffered-SDS followed by SDS-Urea (4% SDS plus 8 M urea).

### PS1xAPP sequential protein extraction

For these experiments, 12-month-old PS1xAPP cortical tissue was used. The TBS soluble fraction was isolated by ultracentrifugation as described above. For the sequential detergent-based extraction, the ultracentrifugation pellets were sequentially extracted using 2% CHAPS (in TBS), RIPA buffer, 2% SDS and finally 4% SDS-8 M urea. For each detergent treatment pellets were resuspended in the appropriate media, incubated for 30 min at 4°C and centrifuged at 30,000×g (30 min, 4°C). All fractions were stored at −80°C for further use.

### Western blots

Western blots were performed as described previously [9], [10], [19], [22]. Briefly, for Abeta peptides, 10–20 µg (unless stated in text) of protein from the different samples were loaded on 16% SDS-Tris-Tricine-PAGE and transferred to PVDF (Immobilon-P Transfer Membrane, Millipore). For sAPPalpha, LC3 and ATPsynthase, 12% SDS-Tris-Glicine-PAGE was used and transferred to nitrocellulose (Optitran, GE Healthcare Life Sciences).

Membranes were then blocked using 2% low-fat milk in TPBS (0.1% Tween-20, 137 mM NaCl, 2.7 mM KCl, 10 mM phosphate buffer_,_ pH 7.4) and incubated overnight, at 4°C, with the appropriate antibody. The membranes were then incubated with anti-mouse or anti-rabbit horseradish-peroxidase-conjugated secondary antibody (Dako) at a dilution of 1/10,000. The blots were developed using the Pierce ECL 2 Western Blotting Substrate detection method (0.5 pg, lower limit sensitivity; Thermo Scientific). The images were obtained with the luminescent image analyzer Image Quant LAS4000 mini (GE Healthcare Life Sciences) and then analyzed using PCBAS program.

### RNA extraction, retrotranscription and real-time RT-PCR

Total RNA was extracted from cells (line N13) using Tripure Isolation Reagent (Roche). After isolation the RNA integrity was determined, by agarose gel electrophoresis, resulting comparable in all samples. Total RNA was determined by spectrophotometric measures.

Retrotranscription (RT) was performed using 4 µg of total RNA as template and High-Capacity cDNA Archive Kit (Ref.4368813, Applied Biosystems) following the manufacturer recommendations. For real time RT-PCR, 40 ng of cDNA were mixed with 2x Taqman Universal Master Mix (Ref.4369016, Applied Biosystems) and 20x Taqman Gene Expression assay probes (TNFalpha, Mm00443258_m1; IL1beta, Mm00434228_m1; IL6 Mm00446190_m1; GAPDH, Mm99999915_g1; 18s, Mm03928990; beta-actin, Mm00607939_s1; Applied Biosystems). PCR reactions were carried out in 96 well plates using either ABI Prism 7000 or 7900HT sequence detector systems (Applied Biosystems). The RT-PCR conditions included two initial steps of 2 min at 50°C to activate the polymerase followed by 10 min at 95°C, and the subsequent 40 cycles of denaturation (95°C, 15 sec) and annealing (60°C, 1 min). The cDNA levels of the different samples were determined using GAPDH as housekeeper. Similar results were obtained using beta-actin or 18s rRNA (not shown). The amplification of the housekeeper was done in parallel with the gene to be analyzed, showing a similar amount of cDNA in all tested samples. All our data are expressed as the mean of at least three different measures [9], [10], [19], [22].

### Amyloid plaque isolation

Beta-amyloid plaques were isolated as described [18]. PS1xAPP (12 months of age) cortical samples were homogenized using Dounce as above and centrifuged at 2,000×g for 30 min (4°C). The supernatant was further centrifuged for 1 hour at 20,000×g to collect the pellet, enriched in amyloid beta plaques. This pellet was resuspended in PBS, layered over a discontinuous sucrose gradient (0.4 ml of 1.0 M; 0.4 ml of 1.2, 0.8 ml of 1.4 and 0.4 ml of 2.0 M sucrose in PBS, pH 7.4) and centrifuged at 100,000×g (Optima MAX Preparative Ultracentrifuge, Beckman Coulter) for 1 h at 4°C using a TLS-55 swing rotor. Then, fractions (from top) were removed and the 1.4–2.0 M sucrose interphase (enriched in amyloid plaques) was collected and diluted in PBS. The enriched amyloid fraction was centrifuged at 100,000×g for 1 h at 4°C, and the pellet was collected and resuspended in PBS pH 7.4. The sample was separated in two aliquots, one was Dounce homogenated and the other one was subjected to sonication (8 pulses of 15 seconds). The two samples were centrifuged at 100,000×g for 1 h at 4°C, and the supernatant and pellet were collected.

### N2a cultures and cell survival

N2a cells were plated at 25,000 cells/cm2 and cultured in high glucose DMEM (Biowest)/OptiMEM (Gibco) (50–50%) supplemented with 2 mM glutamine (Biowest) and 5% (v/v) foetal bovine serum (Biowest) in the presence of penicillin (Biowest) and streptomycin (Biowest) (100 units/ml and 0.01 mg/ml, respectively) at 37°C and 5% CO2. For survival experiments, the medium was changed to high glucose DMEM and serum-deprived. The cells were then treated with different amounts of TBS fractions, from Dounce or sonication homogenization, during 24 hours. The cells survival was assayed by Flow Cytometry using the apoptosis detection kit ANNEXIN V-FITC (Immunostep).

### N13 culture and stimulation

The N13 microglial cells were plated at 15,000 cells/cm2 and cultured in RPMI 1640 (Biowest) supplemented with 2 mM glutamine (Biowest) and 5% (v/v) foetal bovine serum (Biowest), in the presence of 100 U/ml penicillin and 100 µg/ml streptomycin (Biowest) at 37°C and 5% CO2. Cells were treated with different amounts of TBS soluble fractions, from Dounce or sonication, during 3 hours. Then, they were collected to isolate RNA. As controls, N13 cells were treated with equivalent volume of PBS.

### Statistical analysis

Normality of data was first assessed by using Kolmogorov-Smirnov test. Normally distributed data were expressed and represented as mean ± SD. For normally distributed data, means were compared using ANOVA followed by Tukey test (more than two groups) or t-test (for two group comparisons).

## Results

### Low content of soluble Abeta oligomeric in AD patients hippocampal formation

We have first analyzed the Abeta content of soluble fractions prepared from the hippocampal formation (hippocampus proper, subiculum and entorhinal cortex), using Dounce homogenization and ultracentrifugation (see also [9], [10], [19], [22]), of AD (Braak stage V–VI) and non-demented control (Braak stages II to IV) age-matched autopsy samples.

**Figure 1:**
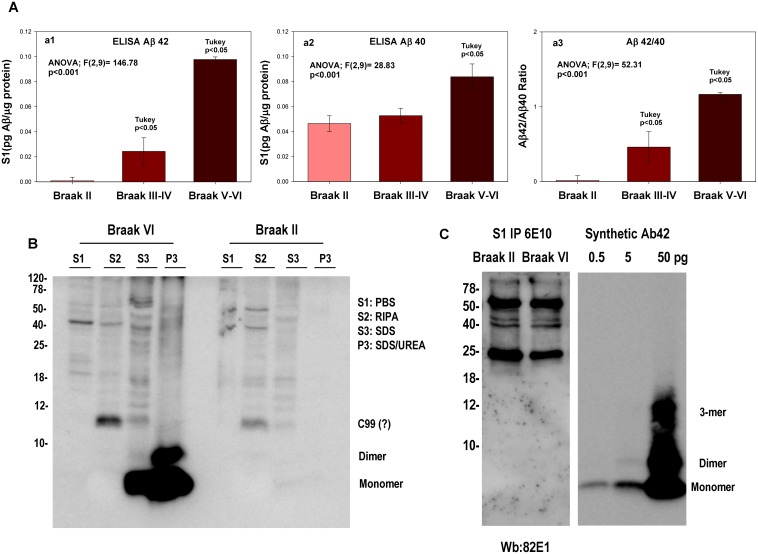
Low soluble Abeta levels were identified in hippocampal samples from age-matched controls (Braak II and III–IV) and demented patients (Braak V–VI). A: Soluble fractions from 11 Braak II, 7 Braak III–IV or 12 Braak V–VI samples were pooled and the Abeta x-42(a1) or x-40(a2) quantified by sandwich ELISA. Each assay was repeated 4 times in triplicate replicas (n = 4 per Braak stage). Although the level of Abeta species from all samples was low (pg/µg protein), there was a significant (showed in the figure) increased in Abetax-42 and Abetax-40 in Braak V–VI. In consequence, there was also an increase in Abeta42/40 ratio (a3) from Braak II to Braak V–VI. B: Western blots of soluble (S1) or detergent-based extracted proteins showed the absence, or below detection limits, of Abeta monomers or oligomers in soluble fractions. Hippocampal samples from Braak VI or Braak II individuals were sequentially extracted in parallel. No Abeta bands were detected in S1 or CHAPS fractions. Monomeric, dimeric and other oligomeric species were clearly identified in 2% SDS and 4% SDS/8M urea samples. C: (left panel) One hundred micrograms of proteins from pooled soluble (S1) Braak II or Braak V–VI fractions were subjected to immunoprecipitation using 6E10 antibody. The immunopellets were analyzed by western blots using 6E10 (not shown) or 82E1. No specific bands, by comparison between Braak II and V–VI, were observed. Control experiments (right panel) demonstrated that 0.5 pg of synthetic Abeta42 could clearly be observed by our western blot system.

Quantitative analysis, using Abetax-42 or Abetax-40 sandwich ELISA (Fig. 1A), demonstrated the presence of very small amounts (pg/µg of protein) of soluble Abeta40-42 peptides in both AD and non-AD samples (see also [4] and [24]). In fact, Braak II samples displayed low levels of Abeta40 whereas Abeta42 was practically undetectable. As shown (Fig. 1A, a1 and a2), we observed a gradual and significant increase in both soluble species, Abeta42 and Abeta40, although more pronounced in the Abeta42 peptide, along the disease progression (3.1-fold in Braak III–IV and 12.5-fold in Braak V–VI). In consequence, the soluble Abeta42 peptide became equimolar with Abeta40 in demented patients (Fig. 1A, a3). Using the Abeta40 plus Abeta42 peptide to estimate the total soluble Abeta species, our soluble samples from AD brains contained approx 4 ng/g tissue of soluble Abeta40-42 peptides. This value was clearly lower to that reported by others [13].

We next attempted to characterize the Abeta species using western blots. Importantly, neither monomeric/dimeric Abeta nor other oligomeric Abeta species could be detected in soluble (S1, Fig. 1B) or in vesiculated (S2, Fig. 1B) Braak VI fractions, as compared with Braak II samples. However, the monomeric-dimeric Abeta (and probably other oligomeric forms) were clearly visible when Abeta plaques were solubilized using 2% SDS (S3) and especially using 4% SDS/8M urea (P3) (Fig. 1B). Furthermore, even using a large amount of proteins (100 µg of soluble fraction, S1) in 6E10 immunoprecipitation assays (Fig. 1C), we were unable to detect any distinctive Abeta specie in Braak V–VI, compared with Braak II.

Taken together, these data are consistent with the existence of low levels of soluble Abeta (pg levels), and the virtual absence of monomeric Abeta in the soluble fraction of AD samples. We cannot discard the presence of minute amounts of monomeric Abeta (below the detection limits of our western blots, 0.5 pg, see Fig. 1C) or small quantities of oligomeric Abeta, not detected by our assays. In any case, these data are in clear contrast with those reported recently [13], [14], [16], [17], [23].

### The homogenization procedure largely determined the content of Abeta species in the soluble fraction

While multiple reasons could explain the different levels of soluble Abeta reported in human AD samples, the initial homogenization of the tissue could be crucial to maintain the integrity of the Abeta plaques. A strong (aggressive) treatment of the tissue could disrupt the aggregated Abeta from plaques and, in consequence, contaminate all fractions with artifactual, plaque-derived, soluble Abeta species. To evaluate this possibility, we have directly compared the effect of homogenization using manual Dounce (see Methods) with sonication at different intensities. As a natural rich source of human Abeta, we have used 12 month-old cerebral cortex from PS1xAPP double transgenic mice. At this age, this model displayed the presence of abundant Abeta plaques in the cerebral cortex (not shown).

**Figure 2:**
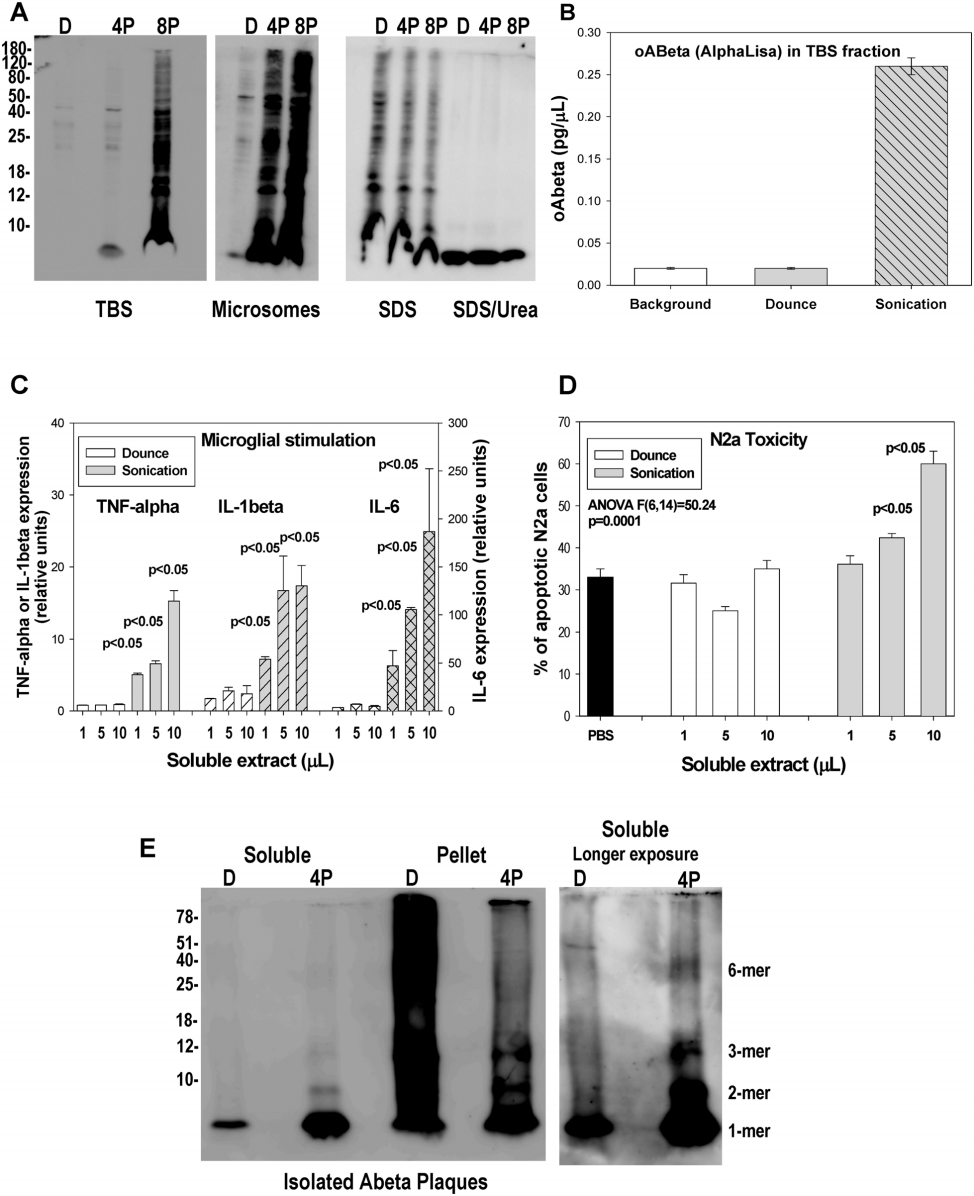
The homogenization procedure has high impact on the integrity of Abeta plaques with the consequent solubilization and redistribution of Abeta in different fractions. A: Western blots, using 82E1, demonstrated the redistribution of monomeric/oligomeric Abeta species in soluble TBS or microsomal fractions (left and middle panel) after homogenization using 4 (4P) or 8 (8P) pulses of sonication (100 watts) as compared with Dounce. This increment was paralleled by a decrease on the Abeta content from 2% SDS or 4% SDS/8M urea extracted samples (right panel). This experiment was repeated four times with similar results. B: The oligomeric Abeta content of TBS soluble fractions was also assayed using an oligomeric (6E10-6E10) specific ELISA assay. No oligomeric Abeta was detected in Dounce prepared TBS fractions, whereas 0.28±0.01 pg/µl of Abeta was detected after sonication of the same cortical tissue. C–D: In vitro experiments demonstrating the stimulation of microglial cultures (C) or the toxic effect (D) of TBS soluble fractions prepared by either Dounce or sonication. A clear dose-dependent stimulation of N13 cells (C) or increase in apoptotic N2a cells (D) was observed exclusively on sonicated samples. E: Sonication could release Abeta from isolated Abeta plaques. Isolated Abeta plaques were homogenized by either Dounce or sonication (4 pulses) and the soluble Abeta release, after ultracentrifugation, was assayed by western blots. As shown, sonication solubilized and released predominantly monomeric and dimeric Abeta. After longer exposures (right panel), other Abeta oligomers, such as trimers or hexamers, could be also identified.

After homogenization, the TBS soluble fraction (equivalent to S1, see above) and the microsomal (equivalent to the dispersible fraction reported by [16], [17]) fractions were isolated by ultracentrifugation (see Methods). The Abeta from plaques were then extracted by sequential solubilization using 2% SDS and 4% SDS plus 8 M urea (following [16], [17] protocol). As expected from our previous work [10], [19], [22] (see Fig. 2A), the monomeric and oligomeric Abeta were preferentially observed in membrane/plaque associated fractions, extracted with 2% SDS and 4% SDS/8M urea. These fractions released a relatively large amount of monomers, dimers and other oligomeric Abeta species. However, as also reported previously [10], [19], [22], in 6–12 months old PS1xAPP mice, these species were absent (or below the detection limit of our western blots) in the TBS soluble fraction or in the microsomal fraction (see Fig. 2A). The lack of oligomeric Abeta in the TBS fraction was also probed by using a 6E10-6E10 oligomeric ELISA (AlphaLisa, Perkin Elmer) assay (Fig. 2B). On the contrary, when the same cortical tissue was subjected to sonication, a pulse-dependent redistribution of Abeta in all soluble fractions was observed. As shown, the TBS soluble fraction was contaminated with relatively high levels of monomeric Abeta (Fig. 2A). Furthermore, we also observed the presence of different putative Abeta oligomers, such as dimers, trimers, and low molecular weight-Abeta in these contaminated soluble fractions (the presence of such oligomers was really high after 8 pulses of sonication, see Fig. 2A). These Abeta oligomers were also clearly detected by 6E10-6E10 oligomeric Abeta ELISA (Fig. 2B). Similarly, the microsomal fractions were also highly contaminated with different Abeta species. Concomitantly, we also observed a decrease in the Abeta content from plaque rich fractions (2% SDS and 4% SDS/8M urea extracted pellets). Therefore, sonication (even using low doses) produced the redistribution of Abeta species from plaques to more soluble fractions.

Since the oligomeric Abeta could act as either, a pro-inflammatory agent, activating microglial cells and producing a classic immunological response [9], or as a cytotoxic agent, producing cell death [10], we next evaluated whether the redistribution of soluble Abeta species, due to the homogenization procedure, was also able to modify the stimulatory and toxic properties of the soluble TBS fractions. As shown in Fig. 2C, different amounts of Dounce-derived soluble fractions induced absolutely no microglial response, as determined by the expression of TNF-alpha, IL-6 and IL-1beta. However, stimulation with the TBS soluble fraction after sonication (4 pulses) produced strong microglial activation, characterized by the induction of the expression of all classic factors assayed. Furthermore, we have also tested the toxicity of the soluble fractions using N2a cells. As expected from previous work [9], the Dounce-derived TBS fraction from 12 month-old PS1xAPP produced no toxicity to N2a cells, whereas sonication-derived fractions produced a clear toxic effect (Fig. 2D).

It could be argued that sonication was indeed releasing highly compartmentalized Abeta, rather than mobilizing Abeta from plaques. Thus, we next directly tested the effect of sonication on isolated Abeta plaques. For these experiments, plaques were isolated by ultracentrifugation on sucrose gradients and homogenized by Dounce or sonication. As shown, mild (Dounce) homogenization released a limited amount of monomeric Abeta from plaques (<1% from total). However, sonication disrupted the Abeta plaques producing (after centrifugation) soluble monomers and, in minor extent, dimeric, trimeric and hexameric Abeta (see Fig. 2E, longer exposure). It is noteworthy the presence of high levels of Abeta oligomers after 4% SDS/8M urea extraction of intact plaques. However, these oligomers were drastically reduced after sonication. Thus, sonication seemed to induce the disaggregation and solubilization of highly aggregated Abeta from plaques. Taken together, these data demonstrated that the homogenization procedure was critical to maintain the integrity of Abeta plaques, and to avoid the contamination of the different fractions with soluble Abeta species.

**Figure 3:**
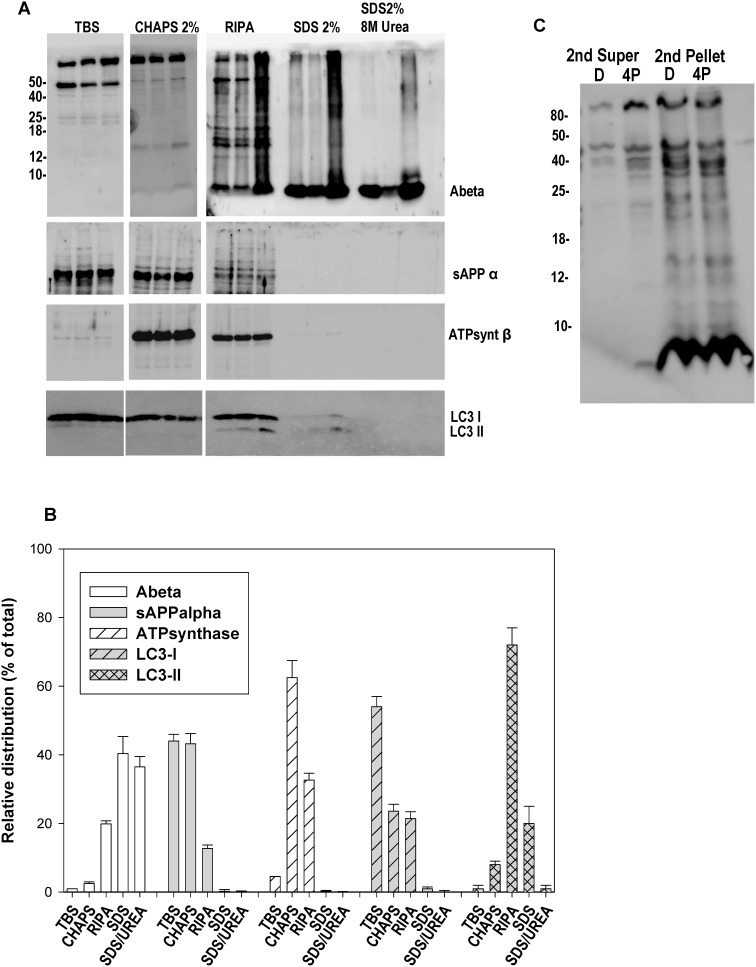
A–B: Biochemical analysis of the Dounce-derived soluble fractions. The presence of APP derived fragments, such as Abeta (6E10) and soluble APPalpha (extracellular), together with LC3-I (cytosolic), ATPsynthase-beta (mitochondrial) and LC3-II (autophagic vesicles) was tested by western blots. A representative western blot (A) or quantitative analysis of three experiments (B) is shown. Although contaminated with cytosolic proteins (LC3-I), the TBS soluble fraction was enriched in extracellular soluble APP-alpha and lacked monomeric Abeta. The monomeric Abeta was predominantly concentrated in vesiculated, LC3-II positive, and plaque associated fractions. **C: Dounce homogenization preserved the integrity of Abeta plaques.** After a first homogenization and centrifugation, pellets containing Abeta plaques were re-homogenized using Dounce or sonication (4 pulses) and the soluble Abeta content assayed using western blots and 6E10. Monomeric Abeta was exclusively observed after sonication.

Next, we evaluated our isolation protocol using (in addition to anti-Abeta) anti-soluble APPalpha antibody (as a marker of the extracellular compartment), anti-ATPsynthase Beta (mitochondria), LC3-I (cytosol) and LC3-II (as marker for autophagic vesicles). First, we determined the distribution of APP-derived soluble peptides (sAPPalpha and Abeta) after serial ultracentrifugation and detergent-based extraction. As expected from previous data, the TBS soluble fraction was enriched on soluble APPalpha and no Abeta was detected (see Fig. 3A and B for quantification). However, this TBS-soluble fraction seemed to be contaminated with cytosolic proteins (detected using LC3-I) and presented low mitochondrial contamination (ATPsynthase). On the other hand, the Abeta species were principally accumulated into the intracellular, vesiculated, LC3-II positive fraction (probably autophagic vesicles, extracted after RIPA treatment) and in plaque enriched fractions (2% SDS and 4% SDS/8M urea releasable fraction). Thus, these data demonstrated that the TBS soluble fractions contained extracellular and cytosolic proteins with low contamination of Abeta plaques.

We have also evaluated to which extent our approach, Dounce homogenization, could release the plaque-associated Abeta from tissue. For this experiment, PS1xAPP cortical samples (12 months of age) were first homogenized, using Dounce, and ultracentrifuged in order to eliminate all putative soluble Abeta. The remaining pellets (containing plaques) were subjected to a second round of homogenization using either Dounce or sonication. Theoretically, all soluble Abeta species release from this second homogenization should derive from intracellular vesicles and/or Abeta plaques. As shown in Fig. 3C, Dounce homogenization did not produce the release of monomeric Abeta whereas monomeric, dimeric and probably trimeric Abeta were clearly observed after sonication of the samples.

## Discussion

In this work, we demonstrate that different conditions of sample homogenization profoundly affect the Abeta content in the soluble fractions, probably by inducing artefactual mobilization of Abeta species from Abeta plaques. We show that using a mild homogenization protocol (manual Dounce), which is the routine procedure in our lab, the soluble fractions from both AD brain samples and PS1xAPP mouse brain tissue contain very small amounts of oligomeric Abeta. In contrast, when using stronger homogenization methods, such as sonication, large amounts of Abeta oligomers were found in the soluble fractions.

We first characterized the soluble Abeta content in hippocampal samples obtained from age-matched non-demented (Braak II, III and IV) and demented (Braak V and VI) human autopsies. The soluble fractions were prepared using mild homogenization conditions, followed by ultracentrifugation, as reported previously [9], [10], [19], [22]. Our data demonstrated the presence of limited amounts of Abeta (pg/µg protein) in the soluble fractions, although we were unable to identify the nature of the Abeta peptides by either western blots or in immunoprecipitation assays using 6E10 or OC (not shown) antibodies. Overall, these data are similar to those reported by some authors [21], [4], but differ from many others reporting the presence of large amounts of different soluble Abeta species, specially in human AD samples [3], [13], [14], [16], [17], [24], [23].

These differences could derive, at least in part, from the different brain regions analyzed and the relative abundance of diffuse versus neuritic Abeta plaques (hippocampal formation with low or non diffuse plaques, unpublished results, versus different cortical areas with relatively high proportion of diffuse plaques; see [13]). However, these conflicting observations could also derive from the different protocols used in the soluble fraction preparation. This observation prompted us to investigate the possible effect of the homogenization protocol in the preparation of soluble fractions and plaque integrity. For this purpose, we directly compared two approaches broadly used for homogenization and isolation of soluble Abeta species, manual Dounce homogenization [9], [4], [21] versus sonication [17], [16]. Using 12-month-old PS1xAPP cortical samples as a source of abundant Abeta plaques, we demonstrate that Dounce homogenization mostly preserves the integrity of Abeta plaques and, in consequence, minimizes the level of contaminating Abeta in soluble fractions, whereas sonication (and probably other strong homogenization approaches) mechanically disrupts Abeta plaques, determining a redistribution of monomeric/oligormeric Abeta in all fractions analyzed. It is particularly relevant the presence of large amounts of Abeta in the soluble TBS fraction and in the microsomal fractions. These two fractions were virtually devoid of Abeta when using manual Dounce homogenization (see also [19]).

A direct consequence of the homogenization method used, is the presence of varying amounts of oligomeric Abeta which strongly influence the pro-inflammatory and toxic effects of the soluble fractions, assayed “*in vitro”*. As we demonstrate in this work, the scarce soluble Abeta, extracted using mild homogenization, produces no microglial stimulation and no degeneration in *“in vitro”* assays. On the contrary, the presence of high levels of monomeric and/or oligomeric species, extracted using sonication, produces clear microglial stimulation and neuronal degeneration. Thus, as a consequence of a strong initial tissue homogenization, the soluble TBS fraction could artifactually become pro-inflammatory and neurotoxic.

It could be argued that the absence of Abeta in the Dounce-homogenized soluble fractions is due to an incomplete disruption of the tissue and that sonication could better mobilize the soluble Abeta pool. Although it is possible that a fraction of the soluble Abeta might indeed remain unextracted in the Dounce-treated tissue, the redistribution observed after sonication most probably reflects the disaggregation of the Abeta plaques. This suggestion is based on: i) the soluble Abeta42 is detected predominantly in samples from demented individuals (Braak V–VI), whereas similar Abeta40 levels are clearly observed in samples with few or no Abeta plaques (Braak II), moderate Abeta pathology (Braak III–IV) and high pathology (Braak V–VI); ii) as showed (see Fig. 2A), sonication produced a redistribution of Abeta from 2% SDS or 4% SDS/8M urea fractions (mostly Abeta plaques) to soluble fractions; iii) we observed a strong effect of sonication treatment on the integrity of biochemically isolated Abeta plaques. In fact, after sonication, the Abeta initially forming SDS-resistant aggregates, sedimentable by ultracentrifugation, is solubilized and redistributed into the TBS fraction. Taken together, these data demonstrate that isolation of soluble Abeta species is highly dependent on Abeta plaque preservation, and that the homogenization procedure is a critical step.

The existence of low levels of Abeta species (below the detection limits of our assays) in TBS soluble fractions is in agreement with data obtained by others using microdialysis [7], . In fact, when the brain tissue is not manipulated, the Abeta levels in the interstitial fluid (ISF) are much lower than that obtained in the TBS extractable fractions [7], [8]. As we have demonstrated in this work, if the integrity of Abeta plaques is preserved during homogenization, the levels of soluble Abeta are very low, below the detection limits of most common approaches. However, when the Abeta plaques are disturbed, more soluble Abeta is clearly detected. Thus, the plaques constituted a very large pool of Abeta (either monomeric or oligomeric) than could be released not only by treatment using detergents or formic acid but mechanically during homogenization using strong conditions (such as sonication).

Based on the above data, it could be argued that soluble fractions isolated using mild (possible low recovery) or strong (partial plaque solubilization) homogenization approaches are not representative of the real Abeta content in the interstitial fluids (see also [8]), and that only microdialysis, using large cutoff membranes, may better represent the actual Abeta equilibrium in the ISF (see [20]). However, taken into account that microdialysis is an *“in vivo”* procedure, the isolation of soluble fractions is the only approach to analyze postmortem human tissue. Thus, to characterize the soluble Abeta species from human samples, special care must be taken in the initial tissue homogenization since, due to the Abeta plaque instability, a strong homogenization procedure could produce a vast redistribution of the Abeta species in all soluble and insoluble fractions. This artifact could explain the dissimilar and somehow controversial data between different groups analyzing human AD samples.
